# Regulation of Human Renal Transporters by Pregnancy-Related Hormones in Primary Proximal Tubular Epithelial Cells

**DOI:** 10.3390/metabo16050292

**Published:** 2026-04-24

**Authors:** Yik Pui Tsang, Kai Wang, Edward J. Kelly, Qingcheng Mao, Jashvant D. Unadkat

**Affiliations:** 1 Department of Pharmaceutics, School of Pharmacy, University of Washington, Seattle, WA 98195, USA; a6245912@uw.edu (Y.P.T.); kaiw95@uw.edu (K.W.); edkelly@uw.edu (E.J.K.);; 2 Kidney Research Institute, University of Washington, Seattle, WA 98104, USA

**Keywords:** renal drug transporters, proximal tubular epithelial cells, pregnancy, pregnancy-related hormones, renal transporter regulation, PBPK modeling

## Abstract

**Background/Objectives**: Pregnancy is associated with increased renal secretory clearance of drugs mediated by organic anion transporters (OATs) and organic cation transporter 2 (OCT2). Circulating concentrations of pregnancy-related hormones (PRHs) increase with gestational age, providing a plausible mechanism for renal OAT and OCT2 regulation. **Methods**: Using primary human proximal tubular epithelial cells (PTECs), we quantified the effects of PRHs, at trimester-specific concentrations, on the mRNA expression of renal drug transporters (apical and basal) and metabolizing enzymes (DMETs), as well as endocytic receptors. PTECs from three female, premenopausal donors were cultured in an optimized Transwell system that maintains measurable OAT activity. PTECs were then exposed for 72 h to trimester-matched PRH cocktails at physiologic (1×) or supraphysiologic (10×) concentrations, with medium replaced every 24 h. DMET and endocytic receptor mRNA were quantified by RT-qPCR, and uptake activities of OAT1/2/3, OCT2, OAT4, and OCTN1 were measured with selective substrates or substrate–inhibitor pairs. **Results**: At 1× PRHs, renal DMET and endocytic receptor mRNA expression was unchanged across trimester-related PRH concentration except for consistent downregulation of PEPT2. Uptake activity for all measured transporters was unchanged. At 10× PRHs, selective changes in mRNA expression of transporters were observed (e.g., induction of OAT1), but these changes did not translate into changes in activity. **Conclusions**: Our data argue against PRHs as the main driver of the increase in OAT-mediated drug secretion during pregnancy. Alternative mechanisms (e.g., flow-dependent mechanotransduction and untested hormones [e.g., prolactin, hCG]) should be evaluated to explain gestation-dependent changes in renal secretory clearance of drugs.

## 1. Introduction

Medication use during pregnancy is widespread. In the U.S., about 89% of more than 30,000 participants reported taking at least one medication during pregnancy, and first-trimester prescription use rose from roughly 37% to 50% over time [1,2]. Population-level registries reported similar findings, with 58% of pregnant people in Sweden and 60% in Norway filling at least one prescription during pregnancy [3,4]. Despite this high prevalence, pregnancy-specific pharmacokinetic (PK), safety, and efficacy data remain sparse, making optimizing drug dosing regimens for the pregnant population a challenge [5].

Pregnancy produces changes in expression and activity of drug-metabolizing enzymes (DMEs) across gestation that alter drug disposition [6,7,8]. Although renal plasma flow and glomerular filtration rate (GFR) increase by ~40–50% by mid-gestation [9], evidence for gestational-age-dependent effects on renal (and hepatic) transporters is limited. Renal proximal tubular epithelial cells (PTECs) express major transporters that mediate vectorial drug secretion. Key transporters include the basolateral organic anion transporters (OAT) 1–3, organic cation transporter 2 (OCT2), and organic anion transporting polypeptide 4C1 (OATP4C1), as well as the apical multidrug-resistance-associated proteins (MRP) 2/4, multidrug and toxin extrusion proteins (MATE) 1/2-K, and P-glycoprotein (P-gp) [10,11,12,13]. Importantly, available data on in vivo renal transporter-mediated secretory clearance of drugs do not cover all trimesters. Therefore, trimester-specific conclusions of changes in drug PK remain provisional. For instance, in the third trimester, digoxin’s unbound renal clearance and renal secretory clearance are nearly doubled compared with values in non-pregnant women, which is consistent with enhanced net tubular secretion through basolateral uptake, plausibly via OATP4C1 [14], and apical P-gp efflux [15]. Oseltamivir carboxylate, an anionic antiviral transported by OAT3, shows lower systemic exposure and higher renal clearance during pregnancy, with the largest proportional increase in the first trimester, which decreases by the third trimester [16,17]. Tenofovir, an OAT1 substrate, shows lower systemic exposure and higher renal clearance during the second and third trimesters [18]. Collectively, these observations point to altered renal transporter activity during pregnancy that is superimposed on the well-documented rise in renal filtration clearance.

We have hypothesized that the elevated plasma concentrations of pregnancy-related hormones (PRHs), including estrogens, progesterone, cortisol, testosterone, and placental growth hormone (PGH), during pregnancy (Table 1) regulate the expression and activity of DMEs and drug transporters (DMETs). For instance, hepatic cytochrome P450 (CYP) 3A is induced about 2-fold in the third trimester, resulting in subtherapeutic plasma concentrations of drugs [15,19,20]. This induction is mainly caused by the elevated plasma concentration of cortisol during pregnancy [21,22,23]. In plated human hepatocytes and HepaRG cells, a trimester-matched PRH cocktail at physiologic concentrations increased the mRNA expression and activity of sodium taurocholate cotransporting polypeptide (NTCP) and OAT2 [21,22]. OATP2B1 mRNA increased modestly, while OCT1 mRNA increased without a clear change in activity. In contrast, OATP1B1/1B3 mRNA expression was downregulated. Hepatic efflux transporters, such as MRP2 and the bile salt export pump, showed modest, system-dependent mRNA changes [21,22]. Despite these in vitro and in vivo data, systematic evaluation of PRH effects on human renal DMETs remains limited. This is in part because maintaining expression and activity of primary PTECs in vitro has been challenging [24,25,26]. We have now overcome this challenge with an optimized Transwell culture system [27]. Here, using this system, we quantified the effects of PRHs, at physiologic concentrations, on the mRNA expression of renal DMETs and endocytic receptors, as well as the activity of the major uptake transporters in primary human PTECs (i.e., OAT1–4, OCT2, OCTN1).

## 2. Materials and Methods

### 2.1. Chemicals and Reagents

Chemicals, reagents, and their suppliers are summarized in Supplementary Table S1.

### 2.2. Isolation and Culture of Primary Human PTECs

Primary human PTECs were isolated, cultured, and characterized as described previously [27]. Disease-free (GFR > 60 mL/min), non-transplantable kidneys from de-identified adult female premenopausal donors were obtained from Organ Procurement Organizations via Novabiosis, Inc. (Durham, NC, USA). Donor authorization for research use was obtained through the organ procurement agency. Because this study used biospecimens from deceased, de-identified donors and the investigators did not receive subject-identifiable information, this work did not constitute human subject research under 45 CFR 46.102(e). Therefore, review by the University of Washington Institutional Review Board was not required. Kidneys were deemed unsuitable for transplantation due to allocation or quality reasons unrelated to intrinsic kidney disease (e.g., not allocated within the clinical time window, prolonged cold ischemia, or donor serologies incompatible with available recipients). Donor information is summarized in Supplementary Table S2. Briefly, kidneys were stored in University of Wisconsin solution at 4 °C until processing (<30 h of cold ischemia time). Cortex tissue was dissected, minced, and enzymatically digested. PTECs were then enriched using sequential isotonic Percoll gradients. Following cell isolation, PTECs were resuspended in defined PTEC medium (DMEM/F-12 containing 1 g/L D-Glucose, 15 mM HEPES, 25 mM NaHCO_3_, 100 ng/mL epidermal growth factor, 15 pM triiodothyronine, 100 nM cortisol (36.25 ng/mL), 1.72 µM insulin, 68.8 nM transferrin, 38.7 nM sodium selenite, 100 U/mL penicillin, 100 µg/mL streptomycin, and 25 µg/mL of amphotericin B) and seeded onto 0.4 µm Transwell inserts coated with 50 µg/cm^2^ of Matrigel (growth factor reduced, phenol red-free) at 2.5 to 3.5 × 10^5^ cells/cm^2^. A83-01 (1 µM) and Y-27632 (10 µM) were added for the first 24 h after seeding to support cell differentiation, survival, and attachment. PTECs were cultured for 3 days in a humidified incubator (37 °C, 5% CO_2_), with daily medium changes, before hormone treatments.

### 2.3. PRH Treatments

Nominal PRH concentrations were based on the geometric means of observed trimester-specific PRH plasma concentrations (Table 1). The PRH cocktail comprised estrone, estradiol, estriol, estetrol, cortisol, progesterone, testosterone, oxytocin, and PGH. All PRHs except for oxytocin and PGH were purchased from Sigma-Aldrich (St. Louis, MO, USA). Oxytocin was purchased MedChemExpress (Monmouth Junction, NJ, USA). Recombinant PGH was purchased from R&D Systems (Minneapolis, MN, USA). PTECs (n = 3 donors) were exposed to PRHs as a cocktail (the “PRH cocktail”) at 1× or 10× their trimester-specific plasma concentrations, in triplicate per donor, for 72 h (37 °C, 5% CO_2_). The 72 h treatment duration was selected so that mRNA expression and transporter activity could be evaluated on a comparable treatment timeline in the same donor-derived PTECs. This duration also allowed more time for transcriptional changes to translate to transporter activity. The latter may occur later than the former because transporter proteins typically have slower turnover than mRNA. The PTEC maintenance medium normally contained 36.25 ng/mL cortisol. During PRH treatments, wells assigned to PRH treatment groups were switched to a cortisol-free medium, and cortisol was supplied only via the PRH cocktail. Therefore, the final cortisol concentration in each group matched the trimester-specific values in Table 1. Treatments were applied to both chambers of the Transwells and were refreshed every 24 h. Following the 72 h treatments, PTECs were harvested for RNA isolation or used in uptake transport assays.

### 2.4. Quantification of the mRNA Expression of Renal DMETs and Endocytic Receptors, PRH Receptors, and Selected Ligand–Receptor Response Genes

At the end of the treatments, total RNA was extracted from PTECs using the PureLink RNA Mini Kit (Thermo Fisher Scientific, Waltham, MA, USA). Samples were treated with DNase I on-column to eliminate genomic DNA and minimize interference with downstream RT-qPCR. RNA concentrations were normalized to the lowest concentration within each donor batch, and RNA was reverse-transcribed into cDNA using the High-Capacity cDNA Reverse Transcription Kit (Thermo Fisher Scientific, Waltham, MA, USA). RT-qPCR was performed with TaqMan probes on a QuantStudio 3 real-time PCR system (Thermo Fisher Scientific, Waltham, MA, USA). Full list of TaqMan probes (25 genes of interest) used is provided in Supplementary Table S3. Additional TaqMan assays for PRH receptors and selected ligand–receptor interaction response genes (controls) were also performed; assay IDs are listed in Supplementary Table S3. Each qPCR reaction contained 10 μL of 2× TaqMan Fast Advanced Master Mix, 1 μL of 20× TaqMan probes, and 9 μL of cDNA diluted in RNase-free water. The thermocycling conditions were 20 s at 95 °C, followed by 40 cycles of 1 s at 95 °C and 20 s at 60 °C. Relative mRNA expression to vehicle control was calculated using the 2^−∆∆Ct^ method [50], with *GAPDH* as the reference gene. *GAPDH* remained stable within donors across conditions at a fixed cDNA input for RT-qPCR (Supplementary Figure S1). C_t_ values > 37 were excluded because of poor signal-to-noise ratio.

### 2.5. Quantification of Uptake Transporter Activity Using Selective Transporter Substrates or Substrate–Inhibitor Pairs

Uptake transporter activity was quantified using previously identified selective substrates or substrate-inhibitor pairs [51], which were subsequently validated in this PTEC Transwell platform [27]. Specifically, uptake was measured using 46.1 nM [^3^H]cidofovir for OAT1, 20 nM [^3^H]nicotinic acid plus 25 μM quercetin for OAT2, 5 μM Glycochenodeoxycholic acid sulfate (GCDCA-S) plus 50 μM cyclosporine for OAT3, 1 μM levocetirizine for OAT4, 303 nM [^3^H]atenolol plus 25 μM mitoxantrone for OCT2, and 2.5 μM [^3^H]ergothioneine for OCTN1. For calculation of active uptake, matched wells were incubated with the corresponding target-transporter inhibitor: probenecid for OAT1/3 (200 μM), Bromosulfophthalein (BSP) for OAT2/4 (200 μM), pyrimethamine for OCT2 (200 μM), or ergothioneine for OCTN1 (1 mM). These conditions were applied here to enrich transporter-specific uptake measurements while minimizing confounding from other transport processes (e.g., efflux). 

After PRH treatments, PTECs were washed twice with warm HBSS^+/+^ (basal pH 7.4; apical pH 6.5) to remove residual PRHs and preincubated with inhibitors or vehicle in warm HBSS^+/+^ for 15 min at 37 °C. Preincubation buffer was then replaced with warm HBSS^+/+^ containing the relevant substrate (± inhibitor, as indicated) in the basal chamber (pH 7.4, for OAT1, OAT2, OAT3, and OCT2) or the apical chamber (pH 6.5, for OAT4 and OCTN1). After 15 min of incubation, the buffer was aspirated and cells were washed three times with ice-cold HBSS^+/+^. Methods for downstream analyte quantification have been described previously [51]. Briefly, for radiolabeled substrates (used for OAT1, OAT2, OCT2, and OCTN1), cells were lysed overnight at room temperature in 1 M NaOH and neutralized with 1 M HCl the next day. Lysates were mixed with Ecoscint liquid scintillation cocktail, and radioactivity was measured on a Tri-Carb B3110TR liquid scintillation counter (PerkinElmer, Waltham, MA, USA). For non-labeled substrates (used for OAT3 and OAT4), cells were lysed in ice-cold 100% acetonitrile containing fexofenadine as the internal standard (50 nM for levocetirizine/OAT4 assays; 250 nM for GCDCA-S/OAT3 assays), diluted 1:1 with water, and quantified by liquid chromatography–tandem mass spectrometry (LC-MS/MS) as previously described [51]. Proteins precipitated in each insert were solubilized in 1 M NaOH overnight at room temperature and neutralized with 1 M HCl the next day. For radioactive assays, total protein was quantified from an aliquot of the same neutralized NaOH lysate used for scintillation counting. For non-radioactive assays, total protein was quantified from the neutralized NaOH-resolubilized protein fraction generated from the acetonitrile precipitate. Protein was measured with the Pierce Bicinchoninic acid Protein Assay Kit on a Spark multimode microplate reader (Tecan, Männedorf, Switzerland). Absolute substrate uptake was normalized to the BCA-measured protein mass in the aliquot used for analyte quantification (pmol/mg). Active uptake by transporters (pmol/mg) was calculated within each condition as absolute substrate uptake amount in non-inhibited wells minus uptake in inhibited wells. Relative active uptake to vehicle control was then expressed as the ratio of active transporter uptake in PRH-treated groups to that in vehicle control.

### 2.6. PRH Quantification by ELISA

To assess the depletion of PRH by PTECs in culture, PRH concentrations in apical and basal media collected after the first 24 h of treatment were measured by ELISA according to manufacturers’ instructions (Supplementary Table S1). Estetrol (E4) depletion was not measured as there are currently no commercial ELISA kits available. Medium samples were diluted with the kit-specific assay diluent to fall within the assays’ linear dynamic range (estrone: 31.25–2000 pg/mL; estradiol: 0.61–10,000 pg/mL; estriol: 19.66–12,000 pg/mL; progesterone: 7.8–1000 pg/mL; cortisol: 6.6–4000 pg/mL; testosterone: 3.9–500 pg/mL; oxytocin: 5.9–750 pg/mL; PGH: 0.5–150 ng/mL). Absorbance was read on a Spark multimode microplate reader (Tecan, Männedorf, Switzerland).

Quantitation and curve fitting for all ELISAs were performed according to the manufacturers’ instructions (Supplementary Table S1). Briefly, for competitive ELISA (all PRHs used except for PGH), blank-subtracted optical density at 450 nm (OD_450_) values were used to calculate the percent hormone bound:

$$\%\;bound\;=\;\frac{B\;-\;NSB}{B_{0}-NSB}\;\times 100\%$$

where B_0_ is the mean OD of the zero standard, B is the OD of each calibrator or sample, and NSB is the OD_450_ of non-specific binding wells. Calibrator OD was fitted with a four-parameter logistic model. Sample concentrations were interpolated and multiplied by the corresponding net dilution factors. For sandwich ELISA (PGH), OD_450_ values were blank-subtracted and interpolated to estimate PGH concentrations by a four-parameter logistic model, then scaled by the corresponding net dilution factors.

### 2.7. Statistical Analysis

After pooling the data across donors, statistical comparisons to vehicle-treated controls were performed using two-way analysis of variance (ANOVA) with Dunnett’s multiple-comparison correction. All data analyses were performed on GraphPad Prism 10.2.1 (GraphPad Software, La Jolla, CA, USA).

## 3. Results

### 3.1. Expression of PRH Receptors in Primary Human PTECs

Primary human PTECs expressed mRNA for the major PRH receptors evaluated in this study, including the glucocorticoid receptor (GR, *NR3C1*), progesterone receptor membrane component 1 and 2 (*PGRMC1/2*), androgen receptor (*AR*), estrogen receptor 1 and 2 (*ESR1/2*), growth hormone receptor (*GHR*), and oxytocin receptor (*OXTR*) (Supplementary Figure S2A–C). To further assess PRH pathway responsiveness in PTECs, we quantified receptor mRNA and selected ligand–receptor-interaction-responsive genes following 72 h exposure to trimester-specific PRH cocktails. *NR3C1*, *PGRMC2*, *ESR2*, and *AR* mRNA expression significantly decreased relative to vehicle control across PRH treatment conditions (Supplementary Figure S2D,E,G,H). In contrast, *ESR1*, *PGRMC1*, and *OXTR* mRNA expression remained largely unchanged (Supplementary Figure S2E,G,I). Among the selected ligand-responsive genes, the mRNA expression of *FOS* (estrogen-responsive) and *SCNN1A* (testosterone-responsive) was significantly induced following PRH treatment (Supplementary Figure S2F,H). These results suggest that primary human PTECs express the receptor machinery required to respond to PRHs.

### 3.2. Effect of PRHs on Renal Transporter mRNA Expression in Primary Human PTECs

**Figure 1:**
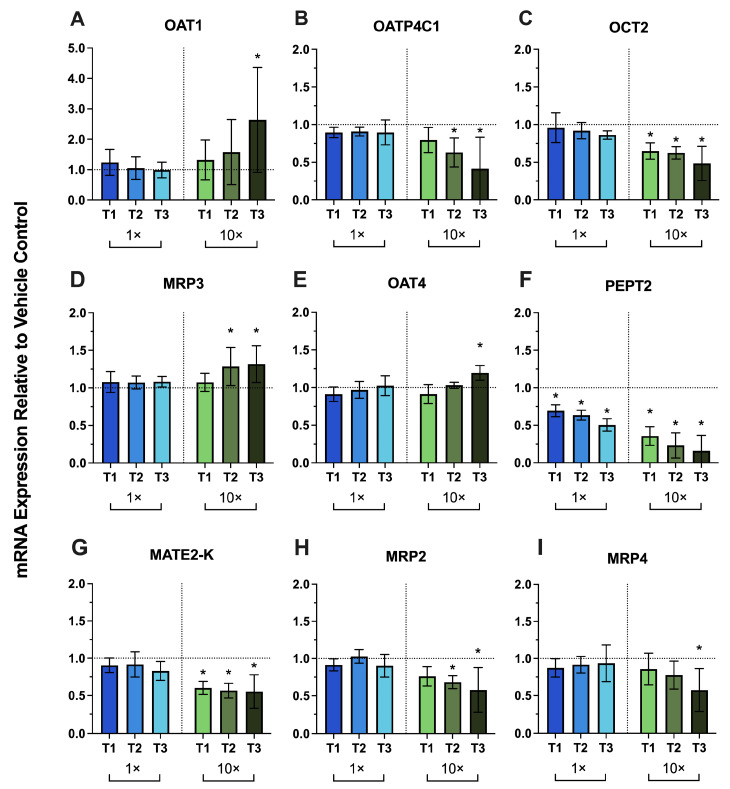
Effect of trimester-specific PRH cocktails (1×, blue bars and 10×, green bars) on the mRNA expression of renal transporters in primary human PTECs. PTECs cultured on Transwells were exposed for 72 h to PRH cocktails added to both the apical and basal chambers (medium refreshed every 24 h). The PRH cocktail was formulated to approximate maternal serum/plasma concentrations for each trimester (T1, T2, T3) at two concentrations (1×, physiologic; 10×, supraphysiologic). Panels: basal uptake transporters ((**A**) OAT1, (**B**) OATP4C1, (**C**) OCT2), basal efflux transporter ((**D**) MRP3), apical uptake transporters ((**E**) OAT4, (**F**) PEPT2), and apical efflux transporters ((**G**) MATE2-K, (**H**) MRP2, (**I**) MRP4). mRNA expression was normalized to GAPDH and expressed relative to vehicle-treated controls (horizontal dashed line at y = 1). Data are mean ± SD from three donors, each quantified in triplicate. Statistical significance (* *p* ≤ 0.05) was assessed using two-way ANOVA followed by Dunnett’s multiple comparisons. Other transporters tested were not significantly affected by PRHs (Supplementary Figure S3).

The CVs of vehicle control technical replicates for each donor for RT-qPCR targets are provided in Supplementary Table S4. Exposure of primary human PTECs to trimester-specific PRH cocktails (T1–T3) at physiologic concentrations (1×) produced no significant changes in renal transporter mRNA expression relative to vehicle controls, except for the peptide transporter 2 (PEPT2) (Figure 1). PEPT2 mRNA was consistently downregulated across all PRH concentrations tested (1× T1–T3: 31%, 37%, and 50%, respectively; 10× T1–T3: 64%, 77%, and 84%, respectively) (Figure 1F). The mRNA expression of basal uptake transporters (OAT1–3, OATP4C1, OCT2) was largely unchanged by 1× PRHs across T1–T3 (Figure 1A–C, Supplementary Figure S3). At supraphysiological PRH concentrations, 10× T3 PRHs induced OAT1 mRNA by 163% (Figure 1A). 10× T2 and 10× T3 PRHs downregulated OATP4C1 mRNA by 38% and 52%, respectively (Figure 1B). OCT2 mRNA was consistently downregulated by PRHs at 10× across T1–T3 (by 35%, 38%, and 52%, respectively) (Figure 1C). Basal efflux transporter MRP3 was stable at 1× PRHs but was slightly upregulated by PRHs at 10× T2 (by 28%) and 10× T3 (by 32%) (Figure 1D). Apical uptake transporter OAT4 mRNA expression was also modestly upregulated by PRHs at 10× T3 (by 20%) (Figure 1E). Apical efflux transporter MATE2-K was consistently downregulated by 10× PRHs across T1–T3 (by 40%, 43%, and 45%, respectively) (Figure 1G). 10× T2 and 10× T3 PRHs downregulated MRP2 mRNA by 32% and 42%, respectively (Figure 1H). 10× T3 PRHs downregulated MRP4 by 43% (Figure 1I). mRNA of other transporters quantified was not affected by PRHs (i.e., OAT2, OAT3, MRP1, organic cation/carnitine transporter 1 and 2 [OCTN1/2], sodium glucose cotransporter 2 [SGLT2], and urate transporter 1 [URAT1], BCRP, MATE1, and P-gp).

### 3.3. Effect of PRHs on the Activity of the Major Renal Uptake Transporters in Primary Human PTECs

**Figure 2:**
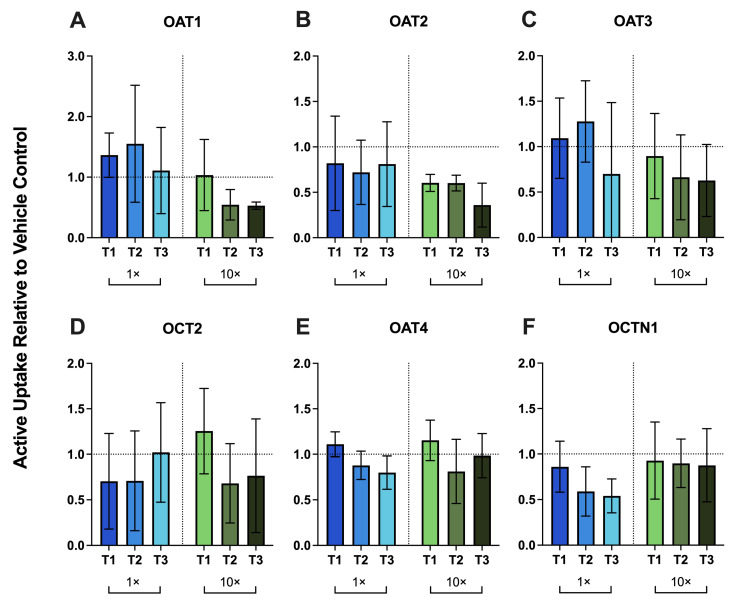
Trimester-specific PRH cocktails (1×, blue bars and 10×, green bars) produced no significant changes in the activity of the major renal uptake transporters in primary human PTECs. PTECs cultured on Transwells were exposed for 72 h to PRH cocktails added to both the apical and basal chambers (medium refreshed every 24 h). The PRH cocktail was formulated to approximate maternal serum/plasma concentrations for each trimester (T1, T2, T3) at two concentrations (1×, physiologic; 10×, supraphysiologic). Activity of uptake transporters ((**A**) OAT1, (**B**) OAT2, (**C**) OAT3, (**D**) OCT2, (**E**) OAT4, (**F**) OCTN1) is presented as the fraction of active uptake relative to vehicle-treated controls (horizontal dashed line at y = 1). Active uptake was calculated as the difference between transporter-selective substrate uptake measured in the absence and presence of inhibitor. Data are mean ± SD from three donors (each quantified in triplicate). Statistical significance was assessed using two-way ANOVA with Dunnett’s multiple comparisons.

The CVs of vehicle control technical replicates for each donor for uptake activity measurements are provided in Supplementary Table S4. Across donors, physiologic (1×) and supraphysiologic (10×) PRH cocktails produced no significant change in uptake activity of OAT1/2/3, OCT2, OAT4, and OCTN1 relative to vehicle (Figure 2A–F). These activity profiles did not mirror the mRNA results shown in Figure 1, where 10× PRHs significantly upregulated OAT1 and OAT4 mRNA and downregulated OCT2 mRNA (Figure 1A–C,E).

### 3.4. Effect of PRHs on Renal DME and Endocytic Receptor mRNA Expression in Primary Human PTECs

**Figure 3:**
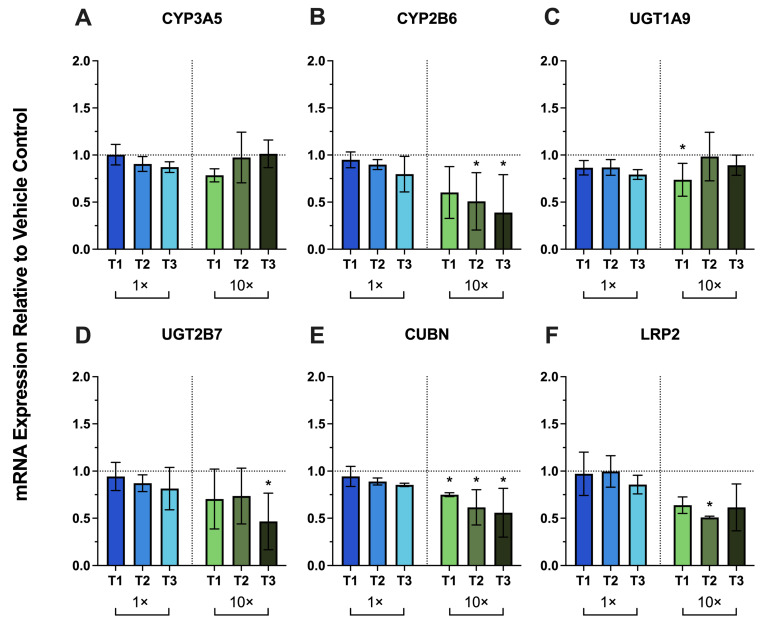
Effect of trimester-specific PRH cocktails (1×, blue bars and 10×, green bars) on the mRNA expression of renal DMEs and endocytic receptors in primary human PTECs. PTECs cultured on Transwells were exposed for 72 h to PRH cocktails added to both the apical and basal chambers (medium refreshed every 24 h). The PRH cocktail was formulated to approximate maternal serum/plasma concentrations for each trimester (T1, T2, T3) at two concentrations (1×, physiologic; 10×, supraphysiologic). Panels: (**A**) CYP3A5, (**B**) CYP2B6, (**C**) UGT1A9, (**D**) UGT2B7, (**E**) cubilin (CUBN), (**F**) megalin (LRP2). mRNA expression was normalized to GAPDH and expressed relative to vehicle-treated controls (horizontal dashed line at y = 1). Data are mean ± SD from three donors, each quantified in triplicate. Statistical significance (* *p* ≤ 0.05) was assessed using two-way ANOVA followed by Dunnett’s multiple comparisons.

Exposure of primary human PTECs to 1× T1–T3 PRH produced no significant changes in the mRNA expression of renal DMEs (CYP3A5, CYP2B6, UDP-glucuronosyltransferase 1A9 [UGT1A9] and UGT2B7) or the endocytic receptors cubilin (CUBN) and megalin (LRP2) relative to vehicle controls (Figure 3A–F). At supraphysiologic PRH concentrations (10×), several genes showed downregulation. 10× T2 and 10× T3 PRHs significantly downregulated CYP2B6 by 49% and 61%, respectively (Figure 3B). UGT1A9 and UGT2B7 were modestly but significantly downregulated by 10× T1 PRHs (33%) and 10× T3 PRHs (53%), respectively (Figure 3C–D). Cubilin was significantly downregulated by 10× T1–T3 PRHs (by 25%, 38%, and 44%, respectively) (Figure 3E). Megalin was significantly downregulated by only 10× T2 PRHs (by 49%) (Figure 3F).

### 3.5. PRH Stability After Exposure to Primary Human PTECs

During the first 24 h of the 72 h PRH exposure, we quantified the stability of each PRH (except estetrol) in the apical and basal media by ELISA. Across hormones and trimesters, measured PRH concentrations closely tracked the corresponding stock concentrations in both chambers, except for cortisol. Estrone, estradiol, estriol, testosterone, oxytocin, and PGH concentration showed no significant loss at 1× or 10× (Supplementary Figure S4A–F,K–P). Cortisol concentration, however, declined by ~50% over 24 h in both apical and basal media across T1–T3 at 1× and 10× (Supplementary Figure S4G–H). Progesterone concentration was largely stable, with a modest but significant ~25% decrease at T3 in the apical compartment (Supplementary Figure S4I–J). No significant apical-basal differences were observed for the remaining PRHs.

## 4. Discussion

In this study, we used an optimized primary human PTEC Transwell model to test whether PRHs alter renal DMET/endocytic receptor mRNA expression and uptake transporter activity. PRH cocktails were formulated to approximate maternal trimester-specific serum/plasma concentrations at physiologic (1×) and supraphysiologic (10×) levels (Table 1). The 10× condition was included to assess potential concentration-dependent effects of PRHs. At 1×, PRHs did not significantly change mRNA expression of renal DMETs or endocytic receptors across donors, except for PEPT2, which was significantly decreased (Figure 1 and Figure 3). Consistent with these mRNA results, 1× PRHs did not significantly change the activity of the major renal uptake transporters (OAT1–4, OCT2, OCTN1) (Figure 2). These results indicate that physiologic concentrations of the PRHs (included in this study) alone are unlikely to account for the increased renal secretory clearance of drugs observed in pregnancy.

A methodological advance in this work was the development of a primary human PTEC Transwell system that sustains measurable OAT mRNA expression and activity. This has been historically a challenge for us and other researchers [24,25]. Our prior work showed that conventionally cultured PTECs on flat plates have no quantifiable OAT transporter activity [25]. Here, we addressed this limitation by optimizing cell isolation and culture procedures. We employed gentle tissue dissociation with density-gradient enrichment of PTECs, which have a buoyant density of approximately 1.036–1.052 g/mL [52]. Additionally, we cultured PTECs on Matrigel-coated Transwell inserts, which provided essential extracellular matrix proteins like laminin and collagen IV. Short pre-culture included Y-27632 and A83-01 to improve PTEC attachment and maintain epithelial phenotype. These improvements to prior protocols, maintained transporter mRNA and activity within a five-day experimental window.

Among genes tested, only PEPT2 was downregulated by physiologic PRH exposure (Figure 1F). PEPT2 is highly enriched at the apical membrane of PTECs and mediates the reabsorption of di/tripeptides and peptide-like drugs (e.g., aminocephalosporins) [53]. Its selective downregulation shown here suggests that the capacity of peptide/peptidomimetic reabsorption could be lower during pregnancy. Because amoxicillin is a PEPT2 substrate [54], downregulation of PEPT2 could contribute to the increased net renal secretory clearance of amoxicillin during pregnancy [55]. Notably, megalin- and cubilin-mediated reabsorption of low molecular weight proteins (e.g., retinol-binding protein, β_2_-microglobulin) is decreased during normal pregnancy [56,57]. PRHs did not reduce megalin or cubilin mRNA in our system (Figure 3E,F). These results suggest that any pregnancy-related attenuation of the endocytosis of low molecular weight proteins may reflect post-transcriptional regulation of megalin/cubilin and/or other potential mechanisms, as discussed later in the Discussion.

At 10× PRH concentrations, selective significant mRNA changes emerged. 10× PRHs downregulated the mRNA of most genes tested, except for the induction of OAT1, OAT4, and MRP3 at 10× T3 (Figure 1). For OAT1, the magnitude of mRNA induction, though significant, was variable across donors (Figure 1). Additionally, mRNA changes in uptake transporters (OAT1, OCT2, OAT4) did not translate into significant changes in uptake activity (Figure 2). We speculate that these results could have at least two non-mutually exclusive explanations. First, there may be post-transcriptional control by PRHs that affects trafficking and turnover of transporters at the plasma membrane. Mechanistically, protein kinase A-dependent SUMOylation and reduced ubiquitination are known to stabilize OAT1/3 at the cell membrane [58]. Conversely, protein kinase C pathways promote ubiquitination and membrane transporter internalization [58]. Second, the 72 h PRH exposure may have been insufficient for modest changes in mRNA expression to translate into measurable changes in transporter activity. Changes in functional transporter abundance at the plasma membrane can lag behind changes in transcript levels because of slower protein turnover and trafficking dynamics [59]. Longer PRH exposure, in a system that maintains quantifiable levels of both transporter mRNA and activity, may therefore be required to detect corresponding changes in transporter activity.

Reporting these largely “negative” findings is important. In hepatocyte systems, PRHs, especially cortisol, were shown to modulate DMET mRNA expression and protein abundance. In sandwich-cultured hepatocytes, HepaRG cells, and Huh7 human hepatocellular carcinoma cells, cortisol was the dominant driver of CYP3A mRNA induction. The combination of PGH, pituitary growth hormone, and cortisol significantly induced CYP3A mRNA expression more than cortisol alone [60,61]. In addition, a T3-matched PRH cocktail induced CYP3A activity to levels comparable to the ~2-fold in vivo increase in the third trimester [60,61]. Hepatic transporters, specifically NTCP, OAT2, and OCT1, are induced by PRHs in plated primary human hepatocytes at the mRNA level [21,22]. By contrast, our results in human PTECs showed no significant PRH effects at physiologic concentrations on renal transporters, suggesting that PRHs are unlikely to be the primary driver of pregnancy-associated increase in renal secretory clearance [55]. This lack of effect was not due to absence of expression of the PRH receptors (Supplementary Figure S2D,E,G,H) or their ability to produce a downstream response after interacting with the PRHs (Supplementary Figure S2F,H). Because the present study focused on transcriptional regulation and transporter activity regulation, protein or activity measurements of renal DMEs was not performed and warrant future study.

Lack of PRH effects at 1× on renal transporters observed in our system suggests that other mechanisms contribute to the higher OAT-mediated secretory clearance during pregnancy. One feature of our experimental design may have reduced our ability to detect PRH-dependent effects. Control PTECs were cultured in maintenance medium containing cortisol at ~36 ng/mL to support cell viability and maintain a proximal-tubule phenotype. In contrast, trimester-matched PRH cocktails contained total cortisol concentrations of 144–313 ng/mL. Thus, both vehicle- and PRH-treated cells may have experienced substantial GR activation, blunting detectable incremental effects of higher cortisol during PRH treatment.

Beyond this limitation of our experimental design, one plausible mechanism that contributes to the increased OAT-mediated renal secretion in vivo is the change in renal hemodynamics during pregnancy. Pregnancy increases GFR (~50%) and renal plasma flow (up to ~80%) [9]. This increases tubular flow and luminal shear stress. Shear stress is a potent regulator of proximal tubule phenotype and can increase OAT1/3 expression and activity [24,62,63,64]. Therefore, flow-dependent mechanotransduction could account for, at least partially, the in vivo increase in OAT-mediated secretion during pregnancy. Future studies should test this hypothesis in microphysiological systems by varying luminal perfusion to model non-pregnant versus pregnant states while quantifying transporter expression and function [24,65].

Although we prioritized a combined trimester-matched PRH cocktail to reflect the physiologic pregnancy hormone milieu, the possibility that individual hormones exert distinct regulatory effects cannot be excluded. Future studies evaluating individual hormones may help identify hormone-specific regulatory effects that were not apparent following exposure to the combined PRH cocktail. Additionally, hormones not tested in this study may also contribute to the renal secretion changes during pregnancy. For instance, circulating prolactin rises across gestation [66]. It signals by activating the prolactin receptor (PRLR), which in turn activates the Janus kinase/signal transducer and activator of the transcription pathway and the phosphoinositide 3 kinase/protein kinase B pathway [67,68]. Because PRLR is expressed in the human kidney [69], prolactin-mediated activation of these pathways may downregulate renal transporters. This is analogous to inflammatory cytokines, which have been shown to dysregulate hepatic and renal transporters [25,70,71,72,73,74]. Another hormone we did not include was human chorionic gonadotropin (hCG). Circulating hCG peaks in T1 and gradually declines thereafter [75]. hCG signals through the luteinizing hormone/choriogonadotropin receptor (LHCGR), which then activates the cAMP/PKA pathway [76]. Because LHCGR has been detected in the human kidney [77], hCG-driven activation of cAMP/PKA could upregulate plasma membrane expression and transport of OAT1/3 by SUMOylation and reduced ubiquitination [58]. Elevation of prolactin/hCG, together with changes in renal hemodynamics, could explain why OAT1/3-mediated secretory clearance peaks early-to-mid pregnancy and returns to the non-pregnant baseline by T3 [78]. These are testable hypotheses for future studies.

## 5. Conclusions

In summary, trimester-matched PRH cocktails at physiologic concentrations did not significantly change renal DMET or endocytic receptor mRNA or uptake activity in primary human PTECs, with PEPT2 downregulation as a reproducible exception. Supraphysiologic PRH exposure produced selective significant mRNA changes without parallel changes in uptake transporter activity. Our data argue against PRHs as the main cause of increased renal OAT-mediated secretion during normal pregnancy. Future work should incorporate additional PRHs (e.g., prolactin and hCG) and employ perfusion-controlled models to test additional mechanistic hypotheses underlying the pregnancy-associated increase in OAT-mediated secretion.

## Figures and Tables

**Table 1:** Geometric mean of the observed steady-state total plasma concentrations (ng/mL) of PRH in the first (T1), second (T2), and third (T3) trimester of pregnancy (adapted from Benzi et al. [22]).

Hormones	T1	T2	T3	References
Estrone	0.811	4.41	9.76	[28,29,30,31]
Estradiol	1.01	6.13	14.0	[28,29,30,31,32,33,34]
Estriol	0.231	2.65	8.68	[28,29,31,35]
Estetrol	—	0.350	0.779	[36,37]
Cortisol	144	267	313	[34,38,39]
Progesterone	26.3	51.7	140	[30,32,33,34,40,41]
Testosterone	0.952	0.952	1.30	[29,30,33]
Oxytocin	0.18	0.23	0.23	[42,43,44,45,46]
PGH	1.9	4.7	12.8	[47,48,49]

## Data Availability

The original contributions presented in this study are included in the article and Supplementary Materials. Further inquiries can be directed to the corresponding author.

## Supplementary Materials

The following supporting information can be downloaded at: https://www.mdpi.com/article/10.3390/metabo16050292/s1, Figure S1: GAPDH cycle threshold (C_t_) stability across donors and conditions; Figure S2: Expression of PRH receptors and selected ligand–receptor-interaction-responsive genes in primary human PTECs; Figure S3: Heatmap summary of PRH effect on mRNA expression of renal drug transporters, drug metabolizing enzymes, and endocytic receptors; Figure S4: PRH stability in apical and basal media over the first 24 h of exposure to PTECs on Transwells (ELISA); Table S1: Chemicals and Reagents; Table S2: PTEC donor demographics; Table S3: TaqMan Assay IDs; Table S4: Coefficients of variation (CV, %) of vehicle control technical replicates for RT-qPCR targets and uptake transporter activity measurements in primary human PTECs.

## Abbreviations

The following abbreviations are used in this manuscript:

ANOVA	Analysis of variance
BCA	Bicinchoninic acid
BCRP	Breast cancer resistance protein
BSA	Bovine serum albumin
BSP	Bromosulfophthalein
cAMP	Cyclic adenosine monophosphate
CAR	Constitutive androstane receptor
cDNA	Complementary DNA
CUBN	Cubilin
CYP	Cytochrome P450
DMEM/F-12	Dulbecco’s Modified Eagle Medium/Nutrient Mixture F-12
DME	Drug metabolizing enzymes
DMET	Drug metabolizing enzymes and transporters
DMSO	Dimethyl sulfoxide
DNase I	Deoxyribonuclease I
EGF	Epidermal growth factor
ELISA	Enzyme-linked immunosorbent assay
4-PL	Four-parameter logistic model
GAPDH	Glyceraldehyde-3-phosphate dehydrogenase
GCDCA-S	Glycochenodeoxycholic acid sulfate
GFR	Glomerular filtration rate
HBSS+/+	Hank’s balanced salt solution with Ca^2+^/Mg^2+^
HBSS−/−	Hank’s balanced salt solution without Ca^2+^/Mg^2+^
hCG	Human chorionic gonadotropin
LC-MS/MS	Liquid chromatography–tandem mass spectrometry
LHCGR	Luteinizing hormone/choriogonadotropin receptor
LRP2	Megalin or low density lipoprotein-related protein 2
MATE	Multidrug and toxin extrusion protein
MRP	Multidrug resistance–associated protein
NSB	Non-specific binding
NTCP	Sodium taurocholate co-transporting polypeptide
OAT	Organic anion transporter
OATP	Organic anion transporting polypeptide
OCT	Organic cation transporter
OCTN	Organic cation/carnitine transporter
OD_450_	Optical density at 450 nm
PBPK	Physiologically based pharmacokinetic
PCR	Polymerase chain reaction
PEPT2	Peptide transporter 2
PGH	Placental growth hormone
P-gp	P-glycoprotein
PK	Pharmacokinetics
PKA	Protein kinase A
PKC	Protein kinase C
PRH	Pregnancy-related hormones
PRLR	Prolactin receptor
PTECs	Proximal tubular epithelial cells
qPCR	Quantitative PCR
RT-qPCR	Reverse transcription quantitative PCR
SGLT2	Sodium–glucose cotransporter 2
SUMO	Small ubiquitin-like modifier
T1	First trimester
T2	Second trimester
T3	Third trimester
UGT	UDP-glucuronosyltransferase
URAT1	Urate transporter 1
