# Supplementary material for: Regulation of Human Renal Transporters by Pregnancy-Related Hormones in Primary Proximal Tubular Epithelial Cells
Source: Metabolites. 2026 Apr 24;16(5):292. doi: 10.3390/metabo16050292 (PMC13208586; doi:10.3390/metabo16050292)
Supplement: Supplementary file 1 [file metabolites-16-00292-s001.zip › metabolites-4210294-supplementary.pdf]

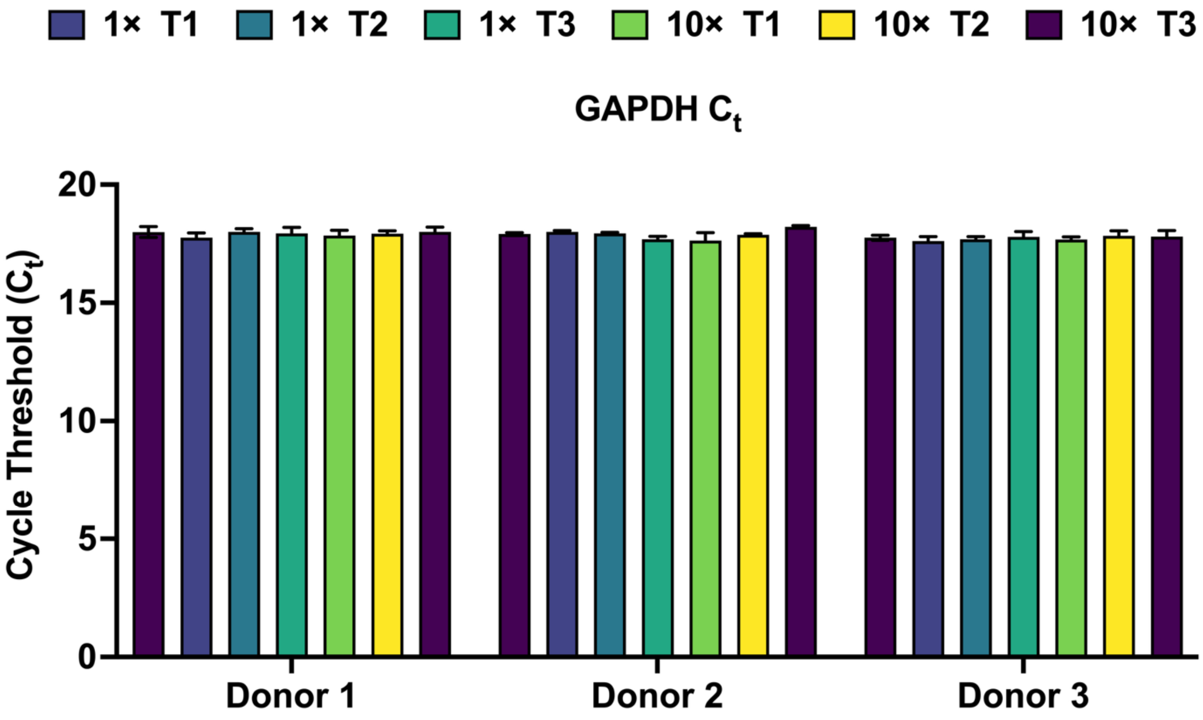

	Donor 1	Donor 2	Donor 3
Mean	17.9	17.9	17.7
Std. Deviation	0.19	0.22	0.17
Std. Error of Mean	0.041	0.048	0.037
Coefficient of variation	1.05%	1.20%	0.96%

Figure S1. GAPDH cycle threshold (C_t) stability across donors and conditions. GAPDH Ct values in primary human PTECs remained stable within donors across pregnancy-related hormone (PRH) treatments for the same amount of cDNA input (90 ng). Therefore, GAPDH was used for mRNA normalization throughout this study. Bars are mean C_t values ± SD of technical triplicates for each PRH condition.

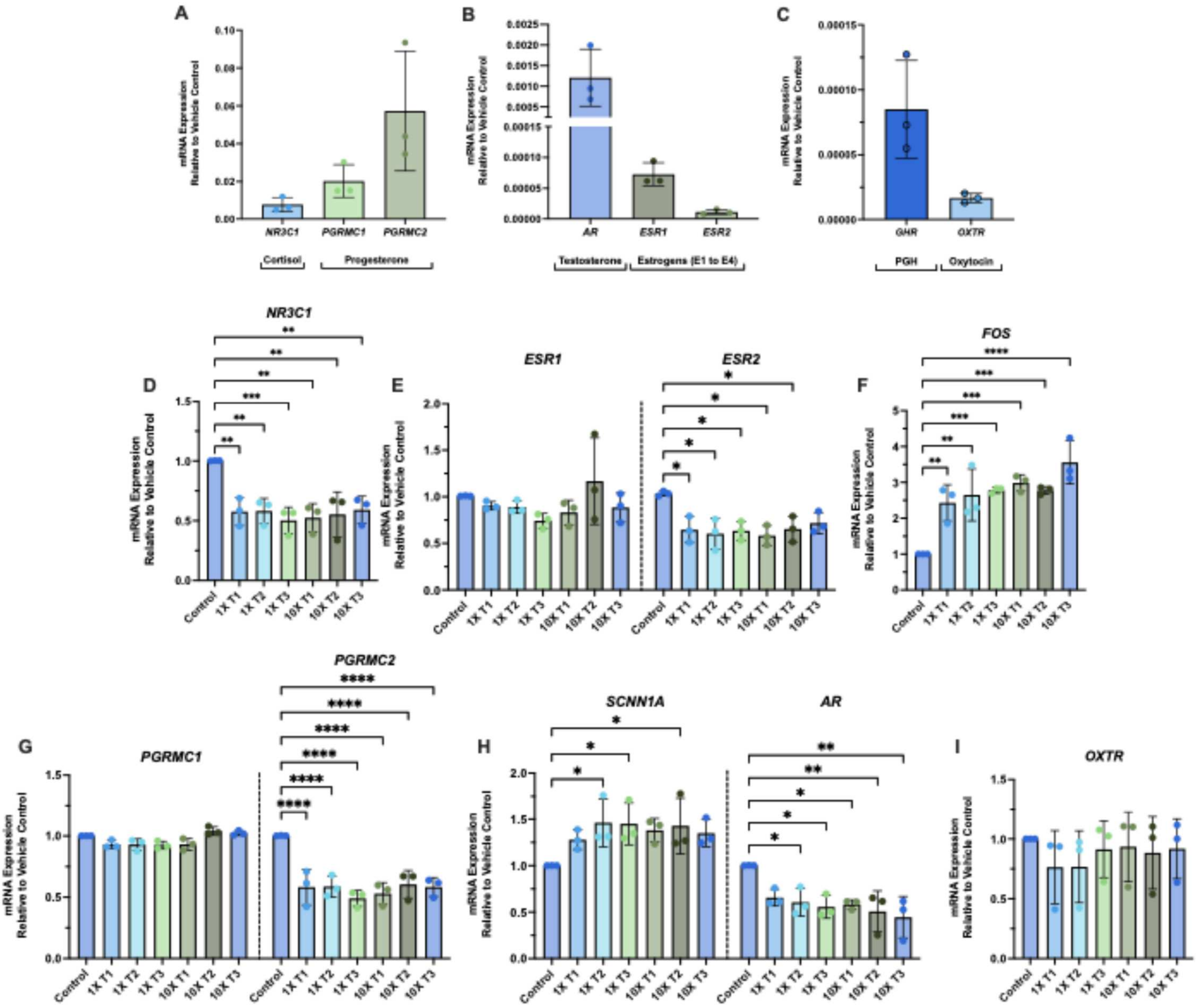

Figure S2. Expression of PRH receptors and selected ligand-receptor interaction responsive genes in primary human PTECs. PTECs cultured on Transwells were exposed to PRH cocktails added to both the apical and basal chambers for 72 h (medium refreshed every 24 h). The PRH cocktail was formulated to approximate maternal serum/plasma concentrations for each trimester (T1, T2, T3) at two concentrations (1×, physiologic; 10×, supraphysiologic). Baseline mRNA expression of the major PRH receptors evaluated in this study is shown in untreated primary human PTECs ([**A**] *NR3C1, PGRMC1*, and *PGRMC2*; [**B**] *AR, ESR1, and ESR2*; [**C**] *GHR and OXTR*). The ligand(s) are listed under each receptor. The effect of trimester-specific PRH cocktails on receptor and selected ligand-receptor interaction responsive gene mRNA expression are shown in the following panels: [**D**] *NR3C1*; [**E**] *ESR1* and *ESR2*; [**F**] *FOS*; [**G**] *PGRMC1* and *PGRMC2*; [**H**] *SCNN1A* and *AR*; [**I**] OXTR. For panels A to C, mRNA expression is shown relative to GAPDH. For panels D–I, mRNA expression was normalized to GAPDH and expressed relative to vehicle-treated controls. Data are mean ± SD from three donors, each quantified in triplicate. Statistical significance in panels D–I was assessed using one-way ANOVA followed by Dunnett's multiple comparisons (*$P < 0.05$, ** $P < 0.01$, *** $P < 0.001$, **** $P < 0.0001$). *NR3C1*, glucocorticoid receptor (GR); *PGRMC*, progesterone receptor membrane component; *AR*, androgen receptor (AR); *ESR*, estrogen receptor; *GHR*, growth hormone receptor; *OXTR*, oxytocin receptor.

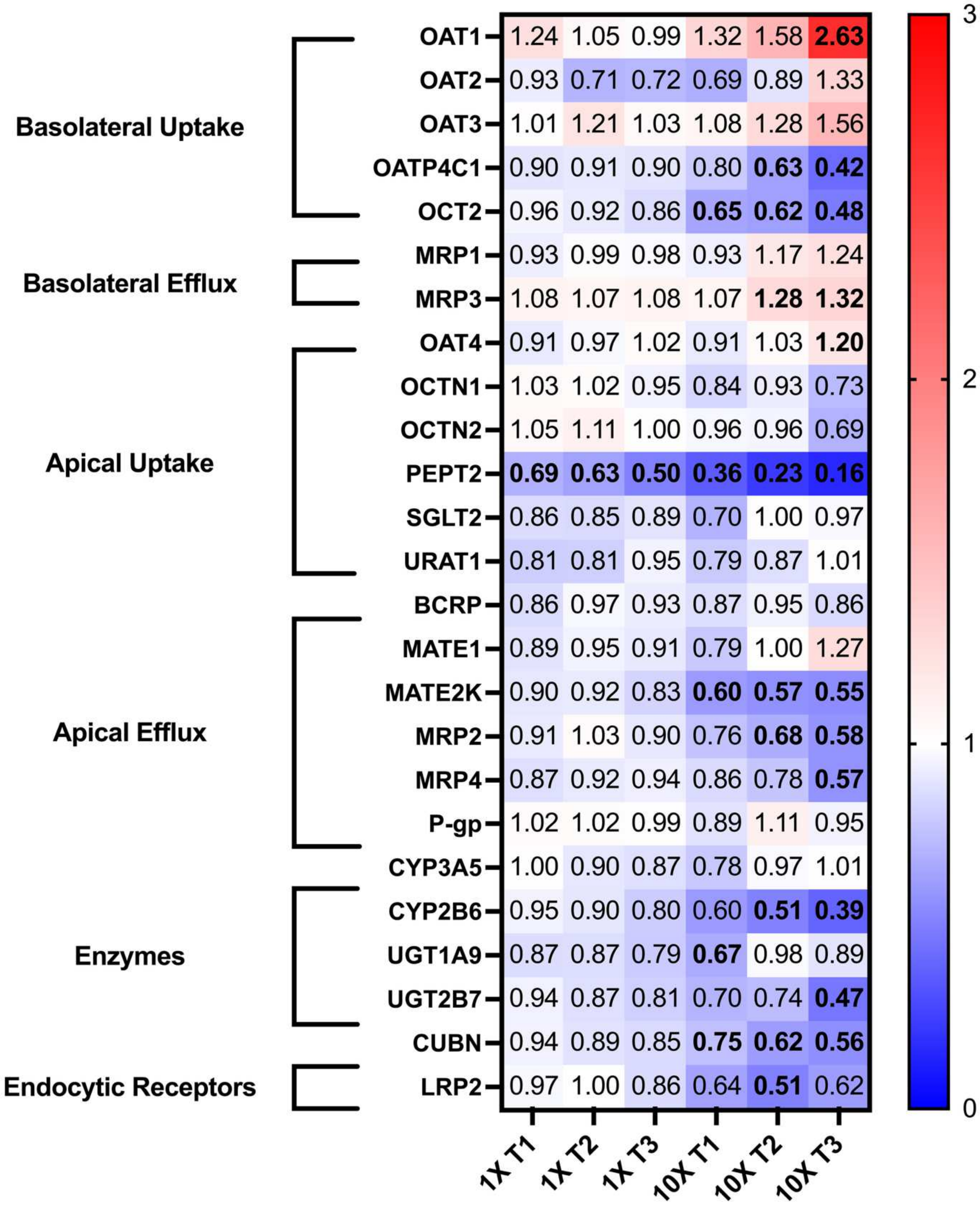

Figure S3. Heatmap summary of PRH effect on mRNA expression of renal drug transporters, drug metabolizing enzymes, and endocytic receptors. PTECs cultured on Transwells were exposed to PRH cocktails added to both the apical and basal chambers for 72 h (medium refreshed every 24 h). The PRH cocktail was formulated to approximate maternal serum/plasma concentrations for each trimester (T1, T2, T3) at two concentrations (1×, physiologic; 10×, supraphysiologic). Heatmap shows average GAPDH-normalized mRNA expression relative to vehicle control (0.1% DPBS) across three donors. Gene expression significantly altered are bolded (*p<0.05, two-way ANOVA with Dunnett's multiple comparison correction).

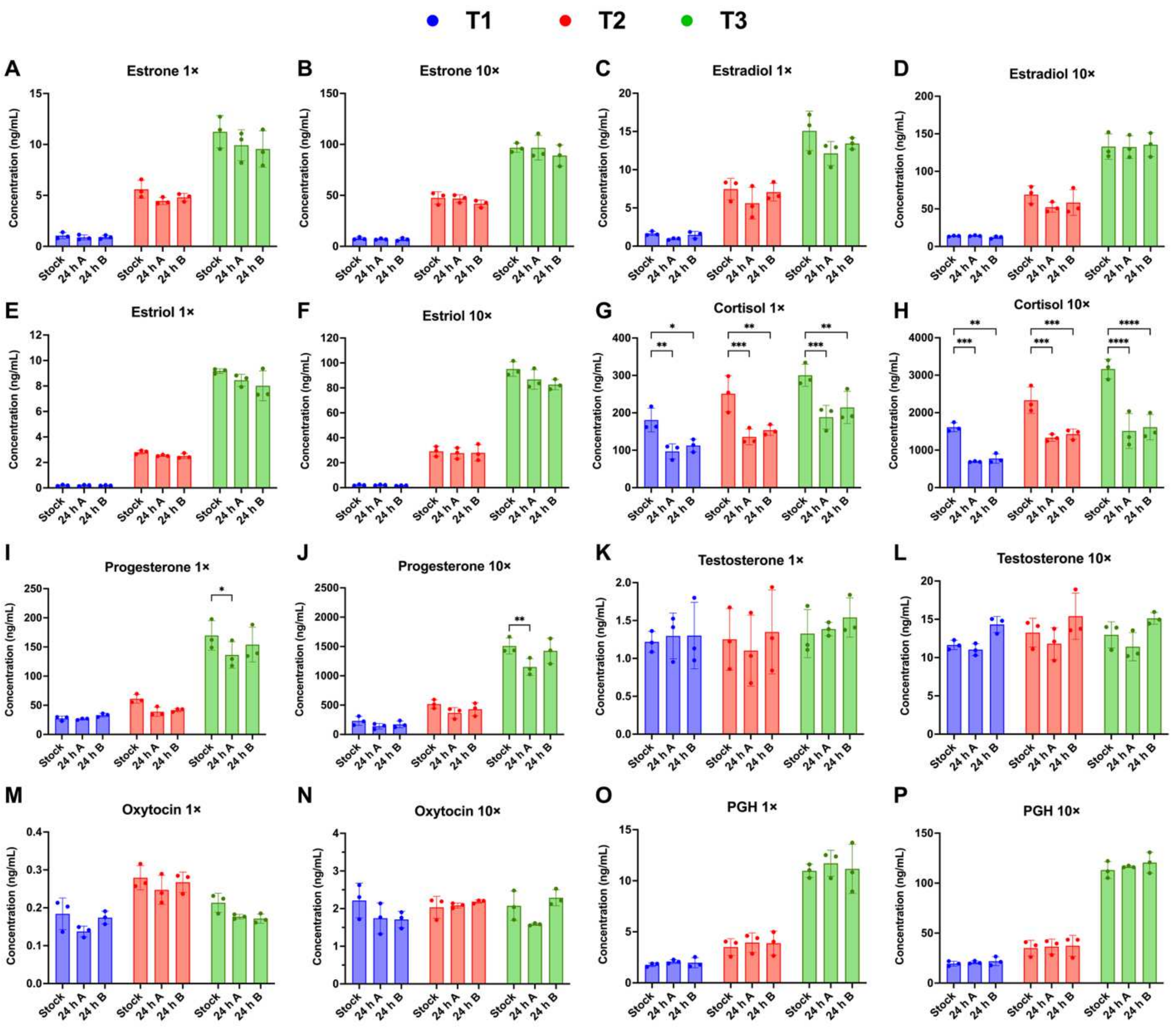

Figure S4. PRH stability in apical and basal media over the first 24 h of exposure to PTECs on Transwells (ELISA). PTECs cultured on Transwells were exposed for 72 h to PRH cocktails added to both the apical and basal chambers (medium refreshed every 24 h). The PRH cocktail was formulated to approximate maternal serum/plasma concentrations for each trimester (T1, T2, T3) at two concentrations (1×, physiologic; 10×, supraphysiologic). Apical and Basal media were sampled at the start of treatment ("stock," immediately after PRH addition) and after 24 h (the first 24 h of a 72 h exposure period; medium was replaced at 24 h). ELISA was used to quantify the concentrations of PRHs ([**A,B**] Estrone; [**C,D**] Estradiol; [**E,F**] Estriol; [**G,H**] Cortisol; [**I,J**] Progesterone; [**K,L**] Testosterone; [**M,N**] Oxytocin; [**O,P**] PGH). Statistical significance was assessed using two-way ANOVA with Dunnett's multiple comparisons (*p≤0.05, **p<0.01, **p<0.001, ****p<0.0001). Cortisol concentrations (1× and 10×), across T1–T3 in both apical and basal chambers, were significantly reduced over 24 h (~50%). T3 progesterone (1× and 10×) concentrations in the apical chamber was significantly, but modestly, reduced over 24 h (~25%). Other PRHs remained largely remained stable over 24 h.

Table S1. Chemicals and Reagents.

Category	Reagent	Supplier
Medium and buffers	Dulbecco's Modified Eagle's Medium/Nutrient Mixture F-12 (DMEM/F-12) powder without glucose	United States Biological (Salem, MA, USA)
	D-(+)-Glucose	Sigma-Aldrich (St. Louis, MO, USA)
	HEPES	
	Sodium bicarbonate ($NaHCO_3$)	
	Sodium hydroxide (NaOH)	
	HBSS with Ca2+ and Mg2+ ($HBSS^{+/+}$)	Thermo Fisher Scientific (Waltham, MA, USA)
	HBSS without Ca2+ and Mg2+ ($HBSS^{-/-}$)	
Enzymes and dissociation reagents	Collagenase type IV (powder)	Thermo Fisher Scientific (Waltham, MA, USA)
	Dispase II (powder)	
	Calcium chloride ($CaCl_2$)	Sigma-Aldrich (St. Louis, MO, USA)
	Ethylenediaminetetraacetic acid disodium salt dihydrate (EDTA) disodium salt dihydrate	
Supplements and additives	Antibiotic–Antimycotic (100×)	Thermo Fisher Scientific (Waltham, MA, USA)
	Bovine serum albumin (BSA)	
	Insulin-Transferrin-Selenium (ITS-G, 100×)	
	Matrigel Growth Factor Reduced (Phenol Red-free)	Corning (Corning, NY, USA)
	HumanKine recombinant human EGF	Proteintech (Rosemont, IL, USA)
	A83-01	MedChemExpress (Monmouth Junction, NJ, USA)
	Y-27632	
	Triiodothyronine	
Solvents and LC–MS reagents	Acetonitrile (LC–MS grade)	Thermo Fisher Scientific (Waltham, MA, USA)
	Dimethyl sulfoxide (DMSO)	
	Formic acid (LC–MS grade)	
Cultureware and plasticware	96-well PCR plates	Thermo Fisher Scientific (Waltham, MA, USA)
	Nalgene Rapid-Flow Sterile Disposable Bottle Top Filters with 0.2 µm PES Membrane	
	Falcon 70 µm cell strainers	Corning (Corning, NY, USA)
	Transwell-Clear Inserts, Polyester (PET) membrane	
Density medium	Percoll density gradient medium	Cytiva (Marlborough, MA, USA)
Pregnancy-related hormones	Estrone	Sigma-Aldrich (St. Louis, MO, USA)
	β-Estradiol	
	Estriol	
	Estetrol	
	Progesterone	
	Hydrocortisone (Cortisol)	
	Testosterone solution (1 mg/mL in acetonitrile)	
	Oxytocin	MedChemExpress (Monmouth Junction, NJ, USA)
	Recombinant Human Growth Hormone 2 (PGH) Protein	R&D Systems (Minneapolis, MN, USA)
RNA isolation, cDNA Synthesis, and RT-qPCR	Dithiothreitol (DTT)	MedChemExpress (Monmouth Junction, NJ, USA)
	96-well PCR and assay microplates	Thermo Fisher Scientific (Waltham, MA, USA)
	PureLink RNA Mini Kit	
	PureLink DNase Set	
	High-Capacity cDNA Reverse Transcription Kit	
	TaqMan Fast Advanced Master Mix	
	TaqMan Gene Expression Assays (Assay IDs in **Supplemental Table S3**)	
	DNase I	
Transporter probes (non-radioactive)	Glycochenodeoxycholic acid-sulfate (GCDCA-S)	LGC Group (Teddington, UK)
	Levocetirizine hydrochloride	MedChemExpress (Monmouth Junction, NJ, USA)
Radioactive transporter probes and scintillation	$[^{3}H]$nicotinic acid (50 Ci/mmol, 20 mM)	American Radiolabeled Chemicals (St. Louis, MO, USA)
	$[^{3}H]$cidofovir (21.7 Ci/mmol, 46.1 mM)	Moravek (Brea, CA, USA)
	$[^{3}H]$atenolol (3.3 Ci/mmol, 0.303 mM)	

	[^{3}H]ergothioneine (0.4 Ci/mmol, 2.5 mM)	
	Ecoscint ORIGINAL	National Diagnostics (Atlanta, GA, USA)
Transporter inhibitors and small molecules	Bromosulfophthalein disodium salt (BSP)	MedChemExpress (Monmouth Junction, NJ, USA)
	Probenecid	
	Pyrimethamine	
	Cyclosporine A	
	Mitoxantrone	
	Quercetin	
	Ergothioneine	
	Fexofenadine hydrochloride	
Protein quantification	Pierce Bicinchoninic Acid (BCA) Protein Assay Kit	Thermo Fisher Scientific (Waltham, MA, USA)
ELISA kits	Estrone Competitive ELISA Kit	Thermo Fisher Scientific (Waltham, MA, USA)
	Estradiol ELISA Kit	Caymen Chemical (Ann Arbor, MI, USA)
	Estriol ELISA Kit	Arbor Assays (Ann Arbor, MI, USA)
	Progesterone ELISA Kit	Caymen Chemical (Ann Arbor, MI, USA)
	Cortisol ELISA Kit	
	Testosterone ELISA Kit	
	Oxytocin ELISA Kit	
	Growth Hormone 2 (PGH) Protein ELISA Kit	MyBiosource (San Diego, CA, USA)

Table S2. PTEC donor demographics.

Donor	Age	BMI (kg/m^2)	Sex	Race	Cause of Death	Alcohol Use	Tobacco Use	Substance Use
Donor 1	43	40.9	Female	White	Anoxia	No	No	No
Donor 2	49	25.5	Female	White	Anoxia	No	No	No
Donor 3	44	25.7	Female	White	Anoxia	Yes	No	No

Table S3. TaqMan Assay IDs.

Gene	Assay ID
OAT1 (*SLC22A6*)	Hs00537914_m1
OAT2 (*SLC22A7*)	Hs00198527_m1
OAT3 (*SLC22A8*)	Hs01056646_m1
OATP4C1 (*SLCO4C1*)	Hs00698884_m1
OCT2 (*SLC22A2*)	Hs01010726_m1
MRP1 (*ABCC1*)	Hs01561483_m1
MRP3 (*ABCC3*)	Hs00978452_m1
OAT4 (*SLC22A11*)	Hs00945829_m1
OCTN1 (*SLC22A4*)	Hs00268200_m1
OCTN2 (*SLC22A5*)	Hs00929869_m1
PEPT2 (*SLC15A2*)	Hs01113665_m1
SGLT2 (*SLC5A2*)	Hs00894642_m1
URAT1 (*SLC22A12*)	Hs01030727_m1
BCRP (*ABCG2*)	Hs01053790_m1
MATE1 (*SLC47A1*)	Hs00217320_m1
MATE2-K (*SLC47A2*)	Hs00945652_m1
MRP2 (*ABCC2*)	Hs00960489_m1
MRP4 (*ABCC4*)	Hs00988721_m1
P-gp (*ABCB1*)	Hs00184500_m1
CYP3A5 (*CYP3A5*)	Hs00241417_m1
CYP2B6 (*CYP2B6*)	Hs03044634_m1
UGT1A9 (*UGT1A9*)	Hs02516855_sH
UGT2B7 (*UGT2B7*)	Hs00426592_m1
Cubilin (*CUBN*)	Hs00153607_m1
Megalin (*LRP2*)	Hs00189742_m1
GAPDH (*GAPDH*)	Hs99999905_m1
SCNN1A	Hs00168906_m1
FOS	Hs00170630_m1
NR3C1	Hs00353740_m1
PGRMC1	Hs00998344_m1
PGRMC2	Hs01128672_m1
AR	Hs00171172_m1
ESR1	Hs01046816_m1
ESR2	Hs01100353_m1
OXTR	Hs00168573_m1

Table S4. Coefficients of variation (CV, %) of vehicle control technical replicates for RT-qPCR targets and uptake transporter activity measurements in primary human PTECs. For each donor, CVs were calculated from technical triplicate vehicle control measurements before donor-level normalization of PRH treatment to the corresponding mean vehicle control.

	**mRNA (CV, %)**		
	**Donor 1**	**Donor 2**	**Donor 3**
**OAT1**	18.40	12.74	18.42
**OAT2**	22.97	4.94	25.47
**OAT3**	15.04	2.87	11.68
**OATP4C1**	7.38	4.00	6.28
**OCT2**	11.79	5.56	8.40
**MRP1**	6.04	4.89	4.96
**MRP3**	5.85	3.30	5.25
**OAT4**	14.40	7.36	21.21
**OCTN1**	7.80	8.10	13.58
**OCTN2**	10.49	2.45	11.65
**PEPT2**	8.25	2.79	6.82
**SGLT2**	3.98	10.09	11.83
**URAT1**	5.69	3.55	5.69
**BCRP**	6.28	2.51	11.10
**MATE1**	12.88	1.74	4.12
**MATE2K**	19.77	8.42	14.47
**MRP2**	13.60	4.47	7.71
**MRP4**	10.50	7.30	8.72
**P-gp**	16.37	3.31	8.87
**CYP3A5**	28.27	8.71	4.06
**CYP2B6**	29.83	3.69	3.69
**UGT1A9**	25.95	2.14	6.92
**UGT2B7**	18.59	5.73	11.15
**CUBN**	3.56	3.30	12.20
**LRP2**	2.66	11.58	11.96
	**Activity (CV, %)**		
	**Donor 1**	**Donor 2**	**Donor 3**
**OAT1**	13.13	22.58	33.42
**OAT2**	21.14	25.76	3.93
**OAT3**	31.78	39.40	28.95
**OCT2**	24.89	33.89	37.54
**OAT4**	10.85	13.27	7.46
**OCTN1**	2.90	10.22	13.89
